# Is routine thromboprophylaxis justified among Indian patients sustaining major orthopedic trauma? A systematic review

**DOI:** 10.4103/0019-5413.80037

**Published:** 2011

**Authors:** Ramesh K Sen, Sujit K Tripathy, Amit K Singh

**Affiliations:** Department of Orthopaedics, Postgraduate Institute of Medical Education and Research, Chandigarh, India

**Keywords:** Thromboprophylaxis, trauma, venous thromboembolism

## Abstract

Venous thromboembolism (VTE) is one of the most common preventable cause of morbidity and mortality after trauma. Though most of the western countries have their guidelines for thromboprophylaxis in these patients, India still does not have these. The increasing detection of VTE among Indian population, lack of awareness, underestimation of the risk, and fear of bleeding complications after chemical prophylaxis have made deep vein thrombosis (DVT) a serious problem, hence a standard guideline for thromboprophylaxis after trauma is essential. The present review article discusses the incidence of DVT and role of thromboprophylaxis in Indian patients who have sustained major orthopedic trauma. A thorough search of ‘PubMed’ and ‘Google Scholar’ revealed 10 studies regarding venous thromboembolism in Indian patients after major orthopedic trauma surgery (hip or proximal femur fracture and spine injury). Most of these studies have evaluated venous thromboembolism in patients of arthroplasty and trauma. The incidence, risk factors, diagnosis and management of VTE in the subgroup of trauma patients (1049 patients) were separately evaluated after segregating them from the arthroplasty patients. Except two studies, which were based on spinal injury, all other studies recommended screening/ thromboprophylaxis in posttraumatic conditions in the Indian population. Color Doppler was used as common diagnostic or screening tool in most of the studies (eight studies, 722 patients). The incidence of VTE among thromboprophylaxis-receiving group was found to be 8% (10/125), whereas it was much higher (14.49%, 40/276) in patients not receiving any form of prophylaxis. Indian patients have definite risk of venous thromboembolism after major orthopedic trauma (except spinal injury), and thromboprophylaxis either by chemical or mechanical methods seems to be justified in them.

## INTRODUCTION

Major orthopedic trauma (which includes spine, hip and pelvi-acetabular fractures; multiple long-bone fractures of the lower extremity) is a compelling risk factor for development of venous thromboembolism (VTE) and its potential sequelae pulmonary embolism (PE)^1^. Thromboprophylaxis by means of chemical and mechanical methods have significantly reduced morbidity and mortality in such patients.^1^ Despite uncertainty about few aspects of VTE, the extensive research on this subject has helped many western surgeons to reach at some concrete conclusion; and as a result, 99% of them use thromboprophylaxis after trauma and elective orthopedic surgeries.^2^ Thromboprophylaxis after trauma is still not widely practiced in India,^3^ the cause of which can be attributed to lack of awareness, underestimation of the problem, fear about thromboprophylaxis complications and most importantly the popular belief among surgeons that Indians have low incidence of deep venous thrombosis (DVT). Contrary to previous belief, most of the recent studies show increasing incidence of VTE among the Indian and Asian population, and it is almost equivalent to that reported in Caucasians.^4^–^17^ Nandi *et al*. while reviewing the Chinese literature found an increasing incidence of VTE among the Chinese population, and they placed the orthopedic surgery of the lower limbs in the high-risk group.^18^ Similarly among the Japanese population, the rate of incidence of VTE after arthroplasty surgeries was found to be increasing over the last four decades, though not equivalent to that in North America and Europe.^19^ In a multinational and multi-ethnic study on Asian population, Piovella *et al*. concluded that the incidence of venographic thrombosis in the absence of thromboprophylaxis after arthroplasty and hip fracture surgery is equivalent to that in the western countries.^8^ Many authors believe that the factors responsible for this changing trend are increase in life expectancy and change in dietary habits (increased fat intake) and lifestyle of the Asian/ Indian population over the last two decades.^4^–^6^,^9^ Nandi *et al*. reported that other than genetic factors, certain acquired traits like dietary habits and lifestyle are important factors responsible for VTE. Most of the studies which have reported very low incidence of DVT in India have been conducted in patients undergoing elective orthopedic surgery and used color duplex for diagnosis.^20^–^22^ In the absence of any study in this population under the high-risk condition of trauma, it is unwise to assume that Indians are genetically protected against VTE after trauma. The reluctance among Indian surgeons to provide thromboprophylaxis to trauma patients may have serious medicolegal implications. In the present scenario, a standard guideline for providing thromboprophylaxis to trauma patients in the Indian subcontinent is urgently needed, which should be practical and acceptable for all. In the present article, the severity of the VTE problem is weighed against the potential complications of thromboprophylaxis, like hemorrhage, wound hematoma, infection, thrombocytopenia and most importantly the cost-benefit ratio. The present review is conducted to answer the frequent question raised by most of the orthopedic surgeons: “Is thromboprophylaxis justified among Indian population after major orthopedic trauma?”

## MATERIALS AND METHODS

A thorough search of ‘PubMed’ with keywords ‘venous thromboembolism,’ ‘Indians,’ ‘trauma’ could not find any article. On searching ’Google Scholar’ with the same key words, 10 references were available that were based on venous thromboembolism in Indian patients after orthopedic trauma surgeries.^11^,^14^,^15^,^17^,^21^,^23^–^27^ Most of these studies have evaluated venous thromboembolism in both patients of arthroplasty and patients of trauma. There is no prospective multicentric studies on VTE after major orthopedic trauma. All the above articles were thoroughly reviewed, and only trauma subgroup of patients (n=1049 excluding the arthroplasty patients) was evaluated regarding the incidence, risk factors, diagnosis and treatment.

## RESULTS

The study designs, fracture types and incidence rates of VTE among thromboprophylaxis-receiving and non–thromboprophylaxis-receiving groups in the Indian studies have been depicted in Table 1. Most of the studies (eight studies, 722 patients) used color Doppler/duplex ultrasonography for diagnosis/ screening of DVT. The two studies (367 patients), which were based on spinal injury, do not recommend thromboprophylaxis. The remaining studies strongly recommended thromboprophylaxis/ screening in all although most of them had less than 10% incidence of DVT. The study of Maini was based only on femoral neck fractures and he had performed bipolar hemiarthroplasty in all these cases. These are included under orthopedic trauma. Among the 276 patients of proximal femur or hip fractures (Maini *et al*., excluded because the article does not provide detail data about the distribution of DVT. It was only a statement by the author that he noticed significant reduction in DVT after he started Heparin therapy in later five years) who had not received any form of chemical or mechanical thromboprophylaxis, 40 patients (14.49%) developed VTE, whereas only 10 (11.5%) out of 115 patients of proximal hip fractures (Maini *et al*. excluded), receiving thromboprophylaxis (either mechanical or chemical), developed VTE.

**Table 1:** Various studies about incidence of deep vein thrombosis in Indian patients after major orthopedic trauma

Studies	Study design	Orthopedic trauma	Diagnostic modalities	Prophylaxis	Incidence (%)	Remarks
Sharma *et al*.,^11^ 2002	Prospective	112 hip fractures	Color Doppler	Nil	19.6 (22/112)	High incidence; advised thromboprophylaxis
Agarwala *et al*.,^14^ 2003	Prospective	18 proximal femoral fractures	Venography	+ LMWH (Dalteparin)	45.4 (5/11) in prophylaxis group; 71.4 (5/7) in non-prophylaxis group	High incidence; advised thromboprophylaxis
Bhan *et al*.,^24^ 2004	Prospective	15 lower-limb fractures + 10 spinal injuries	Doppler ultrasound	Mechanical prophylaxis (lymphavision)	0 (0/25)	Advised mechanical prophylaxis
Agarwala *et al*.,^15^ 2005	Prospective	12 patients with femoral neck fracture	Color Doppler	LMWH (Dalteparin)	0 (0/12)	Advised prophylaxis
Maini *et al*.,^17^ 2006	Retrospective	Femoral neck fractures (271 patients)	NA	Initial 5 years: no prophylaxis; next 5 years: prophylaxis	9.9 (20 in non-prophylaxis group and 5 in prophylaxis-receiving group). No exact data about the number of patients in each group	Significant reduction with prophylaxis
Bagaria *et al*.,^26^ 2006	Prospective	102 proximal femur fractures	Duplex ultrasonography	Nil	6.8 (7/102)	Low incidence; advised thromboprophylaxis in high-risk group
Rajgopalan,^27^ 2007	Prospective	77 femur fractures	Color Doppler ultrasound	LMWH (Dalteparin)	6.49 (5/77)	Advised prophylaxis
Mavalankar *et al*.,^21^ 2007	Prospective	55 patients with lower-limb fractures	Duplex ultrasonography	Nil	10.91 (6/55)	Advised prophylaxis in high-risk patients
Saraf *et al*.,^25^ 2007	Prospective	Acute spinal cord injury (70 patients)	42, color Doppler; 28, venography	Nil	10 (7/70)	Low incidence; however, advised routine screening. Clinical suspicion is inadequate
Agarwal *et al*.,^23^ 2009	Prospective	Acute spinal cord injury (297 patients)	Color Doppler	166 with prophylaxis; 131, no prophylaxis	1.81 (3/166) in prophylaxis group; 3.05 (4/131) in non-prophylaxis group	Low incidence, no significant effect of prophylactic heparin therapy

## DISCUSSION

Limitations due to the scarcity of Indian studies on VTE after major orthopedic trauma have restricted us in formulating a guideline for thromboprophylaxis. Hence the literature available on trauma conditions among the western population was also studied, compared with Indian data and extrapolated to the best of our understanding of the subject. Conclusions were reached by discussion among authors over every point, and finally a protocol for thromboprophylaxis in major trauma patients was designed, with special consideration to Indian population.

### Incidence of VTE in major orthopedic trauma in Indian population

The incidence of VTE and its complications is more in patients undergoing major orthopedic surgery than in those undergoing other surgical procedures^4^. Lee *et al*. (2008) have reported 20.1% incidence of VTE in patients undergoing orthopedic surgery as compared to 30% in those undergoing general surgery.^5^ This was a retrospective review of hospital records, and they agreed that there was a lack of awareness regarding VTE among orthopedicians and reluctance on their part to investigate patients with lower limb symptoms of VTE at their setup. There is lack of prospective multicentric trials on VTE in orthopedic-trauma patients in the Indian population. Among the 10 selected studies that were reviewed, the incidence of VTE was reported to be about 14% after hip or proximal femur fractures and their interventions, without receiving any prophylaxis; whereas it was only 8% when prophylaxis was administered. The data is almost similar to that of Leizorvoicz *et al*.,^4^ who conducted an exhaustive review of literatures and reported 18% incidence of DVT in Asian patients not receiving any thromboprophylaxis after hip fracture surgery. However, these Indian studies are not multicentric and randomized. There is no study on VTE in pelvi-acetabular traumatized patients, and a proper study on spine-injured patients is also not found. None of the studies have evaluated for pulmonary embolism, which may occur in isolation also. The actual severity of the problem is difficult to assess with the present literature, and multicentric studies with inclusion of all major orthopedic trauma (pelvi-acetabular fractures; hip, proximal femur, major long-bone fractures; and spine injuries) would be required for true assessment of VTE.

The severity of VTE among non-resident Indian patients also shows a relatively higher incidence. An increasing incidence of DVT (62.5%) has been reported by Dhillon *et al*. using venography from Malaysia, where a significant percentage of population was of Indian origin.^7^ The incidence of proximal DVT was 12.5%. Patients were investigated for PE only on clinical suspicion, and it was proved in one patient. It was not mentioned whether the clinical criteria included monitoring of oxygen saturation. Three deaths occured before venography could be performed. The cause of death was suspected to be PE in all the cases.^7^

In the largest multicentric, centrally audited venographic trial among Asian patients, Piovella *et al*. found incidence of DVT to be 41% in patients undergoing total hip replacement (THR), total knee replacement (TKR) and hip fracture surgery without prophylaxis.^8^ Incidence of proximal DVT was 10.2%, and 4.4% cases having isolated muscular vein DVT were not included in calculation of DVT incidence rate (total incidence, 45.4%). They pointed out that rate of proximal DVT after TKR was 17.1%, which is on higher side of the range reported from western countries. Some of the 19 centers included in the trial were from India; and on country-wise analysis of data, Indians have not been found to have low risk of VTE.^8^

There is wide variation in incidence of DVT reported from India (0%-71%), mainly due to difference in diagnostic modalities and different indices of suspicion.^5^,^6^ After reviewing the Indian literature [Table 1], we found that Indian patients have also definite risk of DVT after major orthopedic trauma other than spinal injuries.

### Pathogenesis

The pathogenesis of VTE after trauma has not been studied extensively in Indian/Asian literature. Western literature reveals that pathogenesis of VTE in trauma starts immediately after injury, much before the patients present at the hospital. In them, all the three factors mentioned by Virchow (endothelial damage, vascular stasis, hypercoagulability) are present.^28^ Most trauma cases need some kind of surgery, which adds further insult, and the manipulation during surgery can result in dislodgement of preformed thrombus.^29^ A prolonged immobility after trauma or surgery further accelerates the event of thrombus formation. It has been shown that trauma patients have decreased serum level of antithrombin III due to release of tissue factor in circulation, thus favoring coagulation, sometimes in the contralateral limb.^30^,^31^

### Risk factors

Americal College of Chest Physicians (ACCP) recognizes presence of certain factors as high risk for VTE, like malignancy, obesity, estrogen use, past history of VTE, cardiac or respiratory failure, etc.^29^ However, attempts to identify factors that consistently predict risk for VTE in trauma have met with little success, mainly because presence of major trauma itself is a major risk factor. Napolitano *et al*. identified age >60 years, Injury Severity Score >30, length of hospital stay, spinal injury and Trauma and Injury Severity Score >85 as important predictors of VTE.^32^ Geerts *et al*. also identified older age and spinal injury as high risk indicators but found Injury Severity Score to be unreliable.^1^ They also found surgery, blood transfusion and fracture of femur or tibia as important predictors of VTE. They reported DVT rate of 46% in patients younger than 30 years and concluded that all patients sustaining major trauma should be considered at high risk for VTE irrespective of presence or absence of risk factors,^1^ a statement which is supported by other studies as well.^30^ Even ACCP, which in its earlier recommendation advised chemoprophylaxis in trauma patients having one additional risk factor, now recommends routine chemoprophylaxis in major trauma.^29^,^33^

On review of Indian literature, we found many studies correlating several risk factors with development of VTE. Other than systemic problems, several trauma variables like fracture type, immobility, bleeding and operative time have definite impact on development of VTE. Most of the reported literature on VTE is based on lower limb arthroplasty and their interventions, followed by spinal injuries. Bagaria *et al*. in a prospective study of 147 patients undergoing major orthopedic interventions like arthroplasty and proximal femur fracture fixation found immobility of >72 hours, malignancy, obesity, surgical duration of greater than two hours as important risk factors for development of VTE in the Indian population.^26^ In support of this study, Sharma *et al*. (prospective study, n= 112, with fractures around hip joint) also found immobilization for more than four days, presence of more than three risk factors, diabetes mellitus, hypertension, smoking and older age as significant risk factors for DVT.^11^ Contrary to this, Agarwala *et al*. (2003) and Rajgopalan *et al*. found no significant correlation of DVT with age, sex, height, weight, type of anesthesia, type of intervention and postoperative blood loss and transfusion.^14^,^27^ For clarification about impact of these variables on DVT, a large prospective multicentric trial is needed, and this issue still remains debated. However, hip and proximal femur fractures pose definite risk of development of VTE; and in the Indian scenario, spinal trauma patients have lesser risk as compared to western population.^23^,^25^ Among spine-injured patients, Saraf *et al*. found old age and quadriplegia as potent risk factor for this complication.^25^ A recent report by Singh *et al*. describing upper extremity DVT following soft tissue trauma around the shoulder has made most of the orthopedic surgeons rethink about the issue of DVT other than lower extremity fractures and spinal injuries.^34^

### Clinical features of VTE

Clinical signs are insensitive to the diagnosis of VTE.^1^,^20^,^35^,^36^ This is especially true in trauma patients in whom lower limb swelling, pain, chest pain, breathlessness and fever can all occur due to injury *per se*.^37^ Braithwaite *et al*. used a 10% fall in oxygen saturation on pulse oxymetry as clinical evidence of PE^38^; however, it can also be present in other trauma-associated conditions like fat embolism syndrome, massive hemorrhage and lung contusion. The low sensitivity and specificity of clinical signs and low suspicion index for VTE and objective investigations delay the diagnosis of VTE.^5^ Sharma *et al*. found that 22 patients who developed DVT had limb edema, while 60% of the patients who did not have DVT also had limb edema. Pain, positive Homan’s sign, induration and fever were present in 45%, 27%, 8% and 8% of patients with DVT and 24%, 12%, 2% and 6% of cases without DVT, respectively. One clinical feature was present in 18.2%; two, in 72.7%; and three, in 9.1% of patients who had DVT. In the group which did not have DVT, 24%, 28%, 30% and 8% of the patients were with zero, one, two and three clinical features, respectively.^11^ Aggarwal *et al*. and Saraf *et al*. found the clinical signs and symptoms to be totally unreliable and recommended routine screening of all patients with a diagnostic tool.^12^–^14^,^25^

### Diagnostic modality

**Figure 1:**
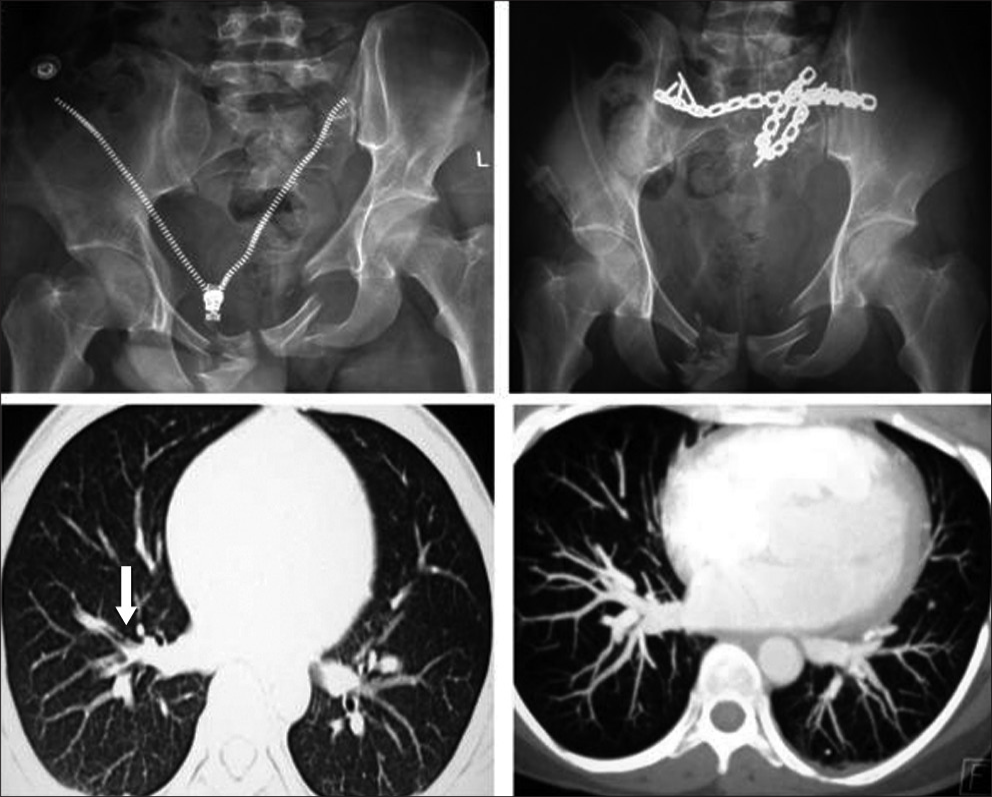
Radiograph showing a vertically unstable pelvic injury in a 22-year-old male. The fracture was stabilized by open reduction and internal fixation with recon plates. Postoperative computerized tomographic pulmonary angiographic films show embolus in right lung field (arrow), compared to normal pulmonary arterial appearance in film on right side.

Incidence of reported VTE varies to a major extent depending on the diagnostic modality used.^5^ There is no single best method for diagnosis.^30^ The common noninvasive diagnostic modalities include compression ultrasonography (USG), real-time B mode compression and color Doppler USG. While duplex ultrasound is most acceptable to surgeons as noninvasive, portable mode, many consider it insensitive, especially in the setting of trauma.^1^,^22^,^30^ Venography is considered gold standard by many investigators; however, it is invasive, cannot be performed if the patient is allergic to contrast has renal failure and it can cause problems due to extravasations of contrast.^30^ Geerts *et al*. found that nearly 30% of venograms in trauma patients yielded inadequate study,^1^ while Montgomery *et al*. found these to give false-negative results in 58% of cases of pelvic thrombi when compared to magnetic resonance venography (MRV).^35^ MRV is very costly; needs transportation of patient to MRI room, which is not always possible; and needs an expert MR radiologist.^30^ Further, many of the small clots detected by MRV may not be clinically significant.^36^ Montgomery *et al*. suggested that venography should be restricted to outcome measures in clinical trials or to patients with inadequate noninvasive screening^30^; clearly, the same applies to MRV as well. Duplex ultrasound has up to 97% sensitivity and 95% specificity^39^; it is noninvasive, repeatable without risk of renal compromise, easily available, portable and has been accepted as the screening method of choice.^2^,^22^,^30^,^40^ It should, however, be remembered that results of ultrasound are user dependent, and a negative screening using noninvasive test does not entirely rule out risk of PE. PE has been reported in 1% to 6% of trauma patients, often developing in patients with a negative screening test.^1^,^30^,^37^ Ventilation-Perfusion scan (V/Q) is the primary diagnostic tool for suspected PE. CT scan and MR angiography are rapidly supplanting V/Q scan, as 52% of V/Q scanning is indeterminate. However, pulmonary angiography remains the gold standard as a diagnostic tool of PE [Figure 1].^41^–^44^ In most of the previous studies color Doppler was used for evaluation of DVT.^11^,^15^,^23^,^25^,^27^ The relative low incidence of DVT in some Indian studies is because of the use of duplex ultrasound as the diagnostic tool.^44^ Nagi *et al*. reported only 8% incidence of DVT by USG, whereas Dhillon *et al*. and Agarwala *et al*. found an increased incidence of DVT by using contrast venography as the diagnostic tool (62.5% and 60%, respectively).^7^,^13^,^44^ Saraf *et al*. found four cases of DVT among 42 patients evaluated by color Doppler; and three among 28 patients evaluated by venography.^25^ They recommended that fibrinogen uptake and D-dimer should be used as screening tests and color Doppler or venography for confirmation of the diagnosis. He also gave importance to the timing of investigations. He attributed this factor as one of the causes for low incidence of DVT in his patients as he performed only a single-time investigation. Agarwala *et al*., while comparing color Doppler with venography for DVT evaluation found sensitivity and specificity of color Doppler duplex sonography as 91.6% and 100%, respectively.^12^ They recommended color Doppler as a reliable and convenient diagnostic tool for DVT evaluation. Thus in the Indian scenario, all patients should be evaluated with color Doppler for DVT. Venography should be reserved for patients with abnormal ultrasound and low clinical probability; or alternatively, for those with a normal ultrasound with a high clinical probability of VTE.^41^,^42^

### Thrombus location

Moser and Lemoine, using a combination of venography, impedance plethysmography, radio fibrinogen scanning, and ventilation perfusion lung scanning, reported no evidence of PE in 21 patients with calf--deep vein thrombosis, whereas more than 50% of patients with proximal thrombosis had lung scan evidence of PE.^45^ Many workers agree that proximal DVT has a higher risk of embolisation causing PE and leading to death.^1^,^30^,^31^,^46^,^47^ Kakkar *et al*. reported PE in about 15% of hospital deaths; and in about 80% of this population, PE contributed very significantly to death.^6^ A recent argument against chemoprophylaxis in Asians/Indians is that most of the thrombi in this population are distal, which are less likely to progress to symptomatic VTE, and most of these resolve without any consequence.^9^,^48^,^49^

Dhillon *et al*. reported a proximal DVT rate of 12.5%; and Piovella *et al*. reported DVT rates of up to 17.1%, which is on the higher side of proximal DVT rates reported from the west (9%-20%).^7^,^8^,^29^ Lee *et al*. have shown that 60% of DVTs in the Indian population are proximal.^5^ Agarwala *et al*. (2002) reported 16.5% of DVTs as proximal, out of which in 12.5% (four out of 24 patients) of cases, the embolus had migrated proximally from calf, and in 4% (one out of 24 patients) there was isolated femoral vein involvement.^12^ Saraf found three (42.8%) cases of proximal DVT out of seven cases of VTE in spinal injury patients, and all of these died because of PE.^25^ Rajgopalan found five (62.5%) cases of proximal and three cases of distal DVT, in which a majority of patients in the study group had femoral fracture interventions.^27^ Bagaria *et al*. reported 3/9 cases of proximal DVT, 5/9 of distal DVT and 1/9 of PE confirmed on necropsy.^26^ Agarwala *et al*. (2003) in another report found 100% and 96.66% of distal DVT in patients receiving and not receiving prophylaxis therapy, respectively. He suggested that though most of the DVTs in the Indian subcontinent appear to be distal in location, these DVTs have high potential for migration and subsequent embolisation.^14^

Unlike patients undergoing elective surgery, in trauma patients, thrombogenesis begins in preoperative period and most of the thrombi are proximal.^1^,^30^,^32^,^46^ Although the distal thrombi have a lower risk of embolisation and chronic venous insufficiency, they cannot be considered having no risk. Without prophylaxis they are more likely to propagate proximally, which substantially increases the risk of pulmonary thromboembolism.^50^ In fact, Wang *et al*. have concluded that isolated calf-muscle DVT shows results comparable to major leg-vein DVT in terms of clinical symptoms, late DVT and proximal propagation; they warned that isolated calf-muscle DVT is a significant clinical entity and should be treated accordingly.^9^ The influence of genetic makeup on risk of VTE cannot be ruled out.^51^ However, is this genetic protection sufficient in the setting of major trauma, wherein every patient is at high risk irrespective of presence or absence of other risk factors?

Most authors found a relatively higher incidence of proximal DVT in the Indian population except Agarwala *et. al*. and none of the studies suggested distal DVT to be a benign entity but advised to treat it aggressively if noticed.^14^,^27^

### Prevention of VTE

Before formulating a guideline for thromboprophylaxis therapy in VTE in the Indian population, the recommendations of American College of Chest Physicians (ACCP) and American Academy of Orthopedic Surgeons (AAOS) should be discussed in brief. The ACCP guidelines on prevention of VTE are prepared by a multidisciplinary, international group of clinician-methodologists, including orthopedic surgeons. ACCP recommendations are based primarily on the results of randomized trials in which VTE (symptomatic and asymptomatic) and bleeding are the primary outcomes [Table 2].^33^ In addition to extensive formal peer review, the primary principle of the ACCP guidelines is to have a transparent link between the evidence and the graded recommendations. The major differences between the two guidelines are that the ACCP lays greater emphasis on DVT prevention and aggressive anticoagulation, whereas AAOS emphasizes fatal-PE prevention over DVT prevention and it has stratified the thromboembolic disease risk and bleeding risk.^33^ Because of the paucity of Indian studies in non-arthroplasty conditions, our view on the treatment or prevention of DVT is based on the western literature and the above two guidelines. However, multicentric randomized control trials with and without prophylaxis are needed for better clarification about DVT prophylaxis.

**Table 2:** ACCP guidelines for venous thromboembolism prophylaxis in hip fracture surgery, lower-extremity fractures, major trauma, and acute spinal cord injury

3.4 ACCP guidelines for hip fracture surgery 3.4.1. For patients undergoing Hip Fracture Surgery (HFS), we recommend routine thromboprophylaxis using fondaparinux (grade 1A), LMWH (grade 1B), adjusted dose Vitamin K Antagonist (VKA) (INR target, 2.5; INR range, 2.0-3.0) [grade 1B], or LDUH (grade 1B). 3.4.2. For patients undergoing HFS, we are against the use of aspirin alone (grade 1A). 3.4.3. For patients undergoing HFS in whom surgery is likely to be delayed, we recommend that thromboprophylaxis with LMWH or LDUH be initiated during the time between hospital admission and surgery (grade 1C). 3.4.4. For patients undergoing HFS who have high risk of bleeding, we recommend the optimal use of mechanical thromboprophylaxis (grade 1A). When the high bleedingrisk decreases, we recommend that pharmacologic thromboprophylaxis be substituted for, or added to, the mechanical thromboprophylaxis (grade 1C). 3.5. Other thromboprophylaxis issues in major orthopedic surgery 3.5.1. Commencement of thromboprophylaxis 3.5.1.1. For patients receiving LMWH as thromboprophylaxis in major orthopedic surgery, we recommend starting thromboprophylaxis either preoperatively or postoperatively (grade 1A). 3.5.1.2. For patients receiving fondaparinux as thromboprophylaxis in major orthopedic surgery, we recommend starting the drug either 6 to 8 h after surgery or the next day (grade 1A). Screening for deep vein thrombosis before hospital discharge 3.5.2. For asymptomatic patients following major orthopedic surgery, we are against the routine use of Doppler ultrasound (DUS) screening before hospital discharge (grade 1A). 3.5.3.4. For patients undergoing HFS, we recommend that thromboprophylaxis be extended beyond 10 days and up to 35 days after surgery (grade 1A). The recommended options for extended thromboprophylaxis in HFS include fondaparinux (grade 1A), LMWH (grade 1C) or a VKA (grade 1C). 3.7. Isolated lower-extremity injuries distal to the knee 3.7.1. For patients with isolated lower-extremity injuries distal to the knee, we suggest that clinicians not routinely use thromboprophylaxis (grade 2A).	5.1. Trauma 5.1.1. For all major trauma patients, we recommend routine thromboprophylaxis if possible (grade 1A). 5.1.2. For major trauma patients, in the absence of a major contraindication, we recommend that clinicians use LMWH thromboprophylaxis starting as soon as it is considered safe to do so (grade 1A). An acceptable alternative is the combination of LMWH and the optimal use of a mechanical method of thromboprophylaxis (grade 1B). 5.1.3. For major trauma patients, if LMWH thromboprophylaxis is contraindicated due to active bleeding or high risk for clinically important bleeding, we recommend that mechanical thromboprophylaxis with Intermittent Pneumatic Compression (IPC) or possibly with Graduated Compression Stocking (GCS) alone be used (grade 1B). When the high bleedingrisk decreases, we recommend that pharmacologic thromboprophylaxis be substituted for, or added to, the mechanical thromboprophylaxis (grade 1C). 5.1.4. In trauma patients, we are against routine DUS screening for asymptomatic deep vein thrombosis (DVT) (grade 1B). We do recommend DUS screening in patients who are at high risk for VTE (e.g., in the presence of a spinal cord injury, lower-extremity or pelvic fracture, or major head injury), and in those who have received suboptimal thromboprophylaxis or no thromboprophylaxis (grade 1C). 5.1.5. For trauma patients, we are against the use of an inferior vena cava (IVC) filter as thromboprophylaxis (grade 1C). 5.1.6. For major trauma patients, we recommend the continuation of thromboprophylaxis until hospital discharge (grade 1C). For trauma patients with impaired mobility who undergo inpatient rehabilitation, we suggest continuing thromboprophylaxis with LMWH or a VKA (target INR, 2.5; range, 2.0-3.0) (grade 2C). 5.2. Acute spinal cord injury (SCI) 5.2.1. For all patients with acute SCI, we recommend that routine thromboprophylaxis be provided (grade 1A). 5.2.2. For patients with acute SCI, we recommend thromboprophylaxis with LMWH, commenced once primary hemostasis is evident (grade 1B). Alternatives include the combined use of Intermittent Pneumatic Compression (IPC) and either LDUH (Grade 1B) or LWMH (grade 1C). 5.2.3. For patients with acute SCI, we recommend the optimal use of IPC and/or GCS if anticoagulant thromboprophylaxis is contraindicated because of high bleeding-risk early after injury (grade 1A). When the high bleeding-risk decreases, we recommend that pharmacologic thromboprophylaxis be substituted for, or added to, the mechanical thromboprophylaxis (grade 1C) 5.2.4. For patients with an incomplete SCI associated with evidence of a spinal hematoma on CT or MRI, we recommend the use of mechanical thromboprophylaxis instead of anticoagulant thromboprophylaxis at least for the first few days after injury (grade 1C). 5.2.5. Following acute SCI, we are against the use of LDUH alone (grade 1A). 5.2.6. For patients with SCI, we are against the use of an Inferior Venacaval Filter (IVC) filter as thromboprophylaxis (grade 1C). 5.2.7. For patients undergoing rehabilitation following acute SCI, we recommend the continuation of LMWH thromboprophylaxis or conversion to an oral VKA (INR target, 2.5; range, 2.0-3.0) (grade 1C).

#### Screening vs. prophylaxis

Some authors suggest routine surveillance in all major trauma cases as chemoprophylaxis does not ensure complete protection,^20^ while most favor routine thromboprophylaxis as noninvasive surveillance leaves a significant number of patients at risk of PE.^1^,^52^,^53^ Probably the most balanced approach is to use routine thromboprophylaxis in trauma patients, with selective screening of high-risk patients.^30^,^32^,^33^

#### Mode of prophylaxis

At present, there is little doubt left regarding the best method of prophylaxis under trauma conditions. Mechanical methods (graduated compression stocking, intermittent pneumatic compression, mechanical foot pumps and foot impulse technology) play a definite role in prevention of DVT,^30^,^36^ but when used alone have proved to give insufficient protection in trauma patients.^52^,^54^–^56^ They cannot be used in patients having below-knee injuries, fixators and traction devices, and there is the problem of compliance as well.^54^ However, they are valuable in cases where chemoprophylaxis is contraindicated due to increased risk of bleeding (because of intracranial, abdominal or chest injuries or other reasons); and during early stages post-trauma, when hemodynamic stability has not been achieved and the patient is waiting for surgery.^30^ They may also be used as an adjunct to chemoprophylaxis in trauma patients at high risk of VTE.^30^,^32^,^33^ In the Indian scenario, the stockinet (above-knee stockings) should be applied to the lower limbs in all polytrauma patients unless it is contraindicated (thigh circumference >81 cm/ incontinence) or impractical because of leg injury.^41^,^42^ The effectiveness of these mechanical devices in the Indian population has not been evaluated extensively. Bhan *et al*. proved the beneficial effect of a mechanical device, viz., low-intensity electrical stimulation by lymphavision, in polytrauma patients and after elective surgeries.^24^ The routine use of vena caval filters as advocated by some workers^52^,^55^ is probably an over-aggressive approach, and they should only be used in cases of proven proximal thrombi.^30^ Their routine use cannot be justified, as they are very costly, need high level of expertise and do not protect against recurrent DVT and post-phlebitis syndrome.^30^,^33^

#### Low-dose unfractionated heparin vs. low-molecular weight heparin

Low-dose unfractionated heparin (LDUH) given subcutaneously for prevention of VTE is ineffective in trauma patients,^46^,^56^ although it has got some role in VTE prevention under other conditions.^33^,^56^ This is probably because the thrombogenic stimuli in trauma overwhelm the beneficial effect of unfractionated heparin.^30^ The greater bioavailability, more predictable dose-response characteristics, longer half-life, greater efficacy and a lower incidence of heparin-induced thrombocytopenia with low–molecular weight heparin (LMWH) as compared with those with unfractionated heparin makes it a suitable agent to be used in trauma patients.^30^,^33^ Its superiority over mechanical prophylaxis and unfractionated heparin has been proved in several studies.^37^,^46^,^56^ The protection offered by it is however not absolute,^56^ and it is justified to supplement it with mechanical compression devices and serial screening in trauma patients with high risk of VTE. The risk of bleeding with LMWH is small (2%-5%) and acceptable considering the dramatic decrease in proximal thrombi which are at high risk of embolisation.^30^ However, one must ensure hemodynamic stabilization before initiating chemoprophylaxis, which usually takes 24 to 36 hours from the time of occurrence of injury.^30^,^33^,^57^ Montgomery *et al*. recommend ’stable hemoglobin level’ as a sign of hemodynamic stability; but considering the hemo-concentration which can occur in trauma patients, the authors recommend ensuring stable vitals and urine output before initiation of chemoprophylaxis.^30^,^47^ Several Indian studies (Agarwala *et al*., Maini *et al*., Rajagopalan *et al*.) have evaluated the beneficial effects of LMWH in polytrauma or lower-limb trauma patients and found it to be safe, effective and easily administrable^14^,^17^,^27^. Agarwala *et al*. found the incidence of DVT to be 45.4% in patients receiving prophylaxis therapy and 71.4% in patients not receiving prophylaxis^14^. However, they did not notice any bleeding complication following thromboprophylaxis.

#### Oral anticoagulant

Oral anticoagulants (Vitamin-K Antagonist like warfarin) are not a practical means for thromboprophylaxis in acute trauma because of delayed onset and prolonged action after withdrawal. Montgomery *et al*. advised to start oral anticoagulant after two weeks of injury;^30^ however, we are of the opinion that oral anticoagulants can be safely started in postoperative period if hemodynamic stabilization has been ensured and patient does not require any more surgery. It usually takes four days to act, which is a safe period against postoperative bleeding. As no further surgical intervention is needed, the drug can be continued with INR (International Normalized Ratio) monitoring (INR in between 2 to 3) and dose adjustment. Another advantage of oral anticoagulants is their low cost. The thromboprophylaxis should be continued till the patient can be mobilized.^30^,^33^ Anti-platelet agents like aspirin are not suggested by ACCP/VTE group as a prophylactic measure in hip fractures and multiple trauma conditions.^33^,^41^,^42^ However, American Academy of Orthopaedic Surgeons (AAOS) recommends aspirin in combination with mechanical prophylaxis in high-risk patients of bleeding. We support the AAOS guidelines in such high-risk conditions.

### Treatment of acute VTE

Treatment of acute thromboembolic episode includes a bolus dose of intravenous heparin of 80 mg/kg body weight followed by 18 mg/kg/h infusion.^58^ Some recent studies have reported effectiveness, safety and ease of subcutaneous low-molecular weight heparin in treatment of VTE.^59^,^60^ This strategy also allows many patients of VTE to be managed entirely as outpatients or to be discharged from hospital^60^; however, it should be used with caution in elderly patients with renal impairment, diabetics and those at risk of bleeding.^58^ Use of LMWH in treatment of VTE is increasing in India as well,^5^ but we suggest use of intravenous heparin in hospitalized patients diagnosed to have DVT and all patients with PE. Considering cost as an important issue in India, unfractionated heparin with warfarin should be administered simultaneously for the initial four to five days till the desired International Normalized Ratio (INR) of 2-3 is achieved, and then the unfractionated heparin can be stopped. Subcutaneous LMWH can be used for outpatients diagnosed to have DVT. Patients who are in hospital for DVT per se can be discharged after shifting them to LMWH or weight-based subcutaneous unfractionated heparin.^61^ However, urgent help of a pulmonologist should be sought if there is any suspicion of massive PE, so that effective medical (streptokinase) or surgical (embolectomy) thrombolysis can be instituted.

This study has its own limitations: (1) the review is entirely based on Indian studies, which lack multicentric and randomized control trails; and (2) statistically significant data about VTE after trauma in the Indian population are not available.

Multicentric randomized control clinical studies are required at different aspects of VTE. There is a definite need to increase awareness about VTE among Indian clinicians and surgeons^3^,^5^ Every hospital should have a formal written thromboprophylaxis protocol of its own, as repeatedly advised by ACCP.^33^

## CONCLUSION

In major-trauma patients, we suggest routine chemoprophylaxis using subcutaneous LMWH after hemodynamic stabilization has been ensured as evidenced by stable hemoglobin level, stable vitals and stable urine output. The patients having isolated upper-limb fractures or isolated lower-limb injury should not be put on chemoprophylaxis.^33^ Patients having head injury, incomplete spinal-cord injury or any other contraindication for chemoprophylaxis should be put on mechanical prophylaxis in the form of graduated stockings (aspirin can be added). Both the methods should be used in major-trauma patients having additional risk factors for VTE. Oral anticoagulants (like warfarin) should be started in postoperative period, and chemoprophylaxis should be continued till patient is mobilized adequately.

We do not suggest routine screening; however, patients having clinical suspicion of VTE despite getting chemoprophylaxis should be investigated using color duppler USG. Patients found positive for VTE should be treated with intravenous heparin and warfarin, and their oxygen saturation should be monitored on a pulse oxymeter. Patients with fall in oxygen saturation or with any other signs of PE should be investigated by ventilation perfusion scan or pulmonary angiography, as available. The treatment should be shifted to oral anticoagulants (like warfarin) when INR of 2-3 is achieved for at least 24 hours.^61^ Patients having DVT and no pulmonary embolism can be treated with intravenous heparin or subcutaneous LMWH, depending upon other indications for hospitalization. Intravenous heparin followed by oral anticoagulants for hospitalized patients is advocated because of their time-tested efficacy and cost-effectiveness and because monitoring of INR and Activated Partial Thromboplastin Time can be easily performed in a patient who has to remain hospitalized for reasons other than DVT (excluding PE). We do not advice hospitalization of patients for DVT *per se*, and they can be discharged on subcutaneous LMWH or weight-based subcutaneous unfractionated heparin.^61^ Treatment of a proved thrombotic episode should be continued for three months, with either LMWH or oral anticoagulants.
